# Identification of Novel Therapeutic Targets in Microdissected Clear Cell Ovarian Cancers

**DOI:** 10.1371/journal.pone.0021121

**Published:** 2011-07-06

**Authors:** Michael P. Stany, Vinod Vathipadiekal, Laurent Ozbun, Rebecca L. Stone, Samuel C. Mok, Hui Xue, Takashi Kagami, Yuwei Wang, Jessica N. McAlpine, David Bowtell, Peter W. Gout, Dianne M. Miller, C. Blake Gilks, David G. Huntsman, Susan L. Ellard, Yu-Zhuo Wang, Pablo Vivas-Mejia, Gabriel Lopez-Berestein, Anil K. Sood, Michael J. Birrer

**Affiliations:** 1 Walter Reed Army Medical Center, Washington D.C., United States of America; 2 Massachusetts General Hospital Cancer Center, Massachusetts General Hospital, Boston, and Harvard Medical School, Boston, Massachusetts, United States of America; 3 Cell and Cancer Biology Branch, National Cancer Institute, Center for Cancer Research, National Institutes of Health, Bethesda, Maryland, United States of America; 4 Department of Gynecologic Oncology, M. D. Anderson Cancer Center, Houston, Texas, United States of America; 5 Brigham and Women's Hospital, Harvard School of Public Health, Boston, Massachusetts, United States of America; 6 Living Tumor Laboratory, British Columbia Cancer Agency, Vancouver, British Columbia, Canada; 7 Department of Obstetrics and Gynecology, Division of Gynecologic Oncology, University of British Columbia, Vancouver, British Columbia, Canada; 8 Peter MacCallum Cancer Centre, Melbourne, Victoria, Australia; 9 Department of Pathology, Genetic Pathology Evaluation Centre, Vancouver General Hospital, Centre for Translation and Applied Genomics, British Columbia Cancer Agency and University of British Columbia, Vancouver, British Columbia, Canada; 10 Department of Medical Oncology, British Columbia Cancer Agency - Southern Interior, Kelowna, British Columbia, Canada; 11 The Vancouver Prostate Centre and Department of Urologic Sciences, University of British Columbia, Vancouver, British Columbia, Canada; 12 Department of Experimental Therapeutics, M. D. Anderson Cancer Center, Houston, Texas, United States of America; 13 Department of Cancer Biology, M. D. Anderson Cancer Center, Houston, Texas, United States of America; 14 Center for RNA Interference and Non-Coding RNA, M. D. Anderson Cancer Center, Texas, United States of America; Baylor College of Medicine, United States of America

## Abstract

Clear cell ovarian cancer is an epithelial ovarian cancer histotype that is less responsive to chemotherapy and carries poorer prognosis than serous and endometrioid histotypes. Despite this, patients with these tumors are treated in a similar fashion as all other ovarian cancers. Previous genomic analysis has suggested that clear cell cancers represent a unique tumor subtype. Here we generated the first whole genomic expression profiling using epithelial component of clear cell ovarian cancers and normal ovarian surface specimens isolated by laser capture microdissection. All the arrays were analyzed using BRB ArrayTools and PathwayStudio software to identify the signaling pathways. Identified pathways validated using serous, clear cell cancer cell lines and RNAi technology. *In vivo* validations carried out using an orthotopic mouse model and liposomal encapsulated siRNA. Patient-derived clear cell and serous ovarian tumors were grafted under the renal capsule of NOD-SCID mice to evaluate the therapeutic potential of the identified pathway. We identified major activated pathways in clear cells involving in hypoxic cell growth, angiogenesis, and glucose metabolism not seen in other histotypes. Knockdown of key genes in these pathways sensitized clear cell ovarian cancer cell lines to hypoxia/glucose deprivation. *In vivo* experiments using patient derived tumors demonstrate that clear cell tumors are exquisitely sensitive to antiangiogenesis therapy (i.e. sunitinib) compared with serous tumors. We generated a histotype specific, gene signature associated with clear cell ovarian cancer which identifies important activated pathways critical for their clinicopathologic characteristics. These results provide a rational basis for a radically different treatment for ovarian clear cell patients.

## Introduction

Clear cell ovarian cancer (CCOC) was originally described as a mesonephroma ovarii in 1939 by Schiller due to its similar appearance to renal cell carcinoma [1]. Further studies since that time, has provided evidence that these tumors are of ovarian origin [2], [3], [4], [5], [6] CCOC represents 4–14% of all epithelial ovarian cancers and its clinical behavior differs from that of the other epithelial histotypes [4], [6], [7]. Patients with stage I CCOC have a 27% risk of recurrence [8] with a five year survival rates for of 60% compared with 80%, for serous tumors [8]. Patients with late stage disease also have a poorer prognosis when compared to patients with advanced stage serous ovarian cancer [8]. This likely reflects CCOC's lower rate of response to the traditional platinum/taxane-based chemotherapy, reported to be between 11–45% during first-line treatment [8], [9]. CCOC patients also have higher rates of thromboembolic events when compared to patients with other epithelial ovarian cancer histotypes [10].

Histologically, CCOC cells are “clear” due to the high cytoplasmic glycogen content that is an artifact of H & E staining [11], [12]. CCOC has been found to have ultrastructural similarity to clear cell carcinoma of the vagina and endometrium. This ultrastructural similarity carries over to genetic similarity as well. Zorn et al. found similar gene expression profiles of clear cell tumors of the ovary, endometrium, and kidney with use of an 11,000 probeset array [13]. This genetic overlap, however, did not extend to the comparison of serous and endometrioid histotypes of ovarian and endometrial origin. Another gene expression profile of CCOC using a 7,129 probe set array demonstrated it to be very distinct from the other histotypes [14]. These studies suggest that there are similar pathways that lead to the clear cell histotype regardless of the organ of origin.

In this study, we present the results of the first whole genome expression profiling of microdissected CCOC samples. Gene ontology and pathway analysis identified major activated pathways involved in hypoxic cell growth, angiogenesis, and glucose metabolism. We hypothesized that these pathways might provide a mechanism for the aggressive clinical nature of CCOC. We demonstrate that clear cell cancer cell lines survive better than serous cell lines under hypoxia and glucose deprived conditions and this is in part due to these activated pathways involving HIF1 α and enolase. *In vivo* experiments using patient derived tissues demonstrate that clear cell tumor xenografts are exquisitely sensitive to antiangiogenesis therapy (sunitinib) compared with serous tumors. Combination therapy of sunitinib and RNAi to HIF1α and enolase demonstrates synergistic anti tumor activity. These results provide a rational basis for specific therapy in these patients.

## Materials and Methods

### Ethics Statement

Ovarian cancer tissue specimens were obtained with informed written consent from patients undergoing bilateral salpingoophorectomy at Vancouver General Hospital following a protocol approved by the University of British Columbia Clinical Research Ethics Board, Canada. Specimens used for profiling were collected under the protocols approved by the institutional review boards of the Brigham and Women's Hospital (Boston, MA, USA) and were obtained with informed written consent from the patients. Animal care and experiments were carried out in accordance with the guidelines and approval by the University of British Columbia - British Columbia Cancer Agency Research Ethics Board (UBC BCCA REB) (Number: H04-60131) and M.D. Anderson Cancer Center Institutional Animal Care and Use Committee, USA (IACUC Number: 12-02-18233).

### Tissue specimens, microdissection, RNA isolation and amplification

Ten clear cell ovarian cancer specimens were obtained from the primary tumors of previously untreated ovarian cancer patients at the Brigham and Women's Hospital (Boston, MA). A set of 10 normal ovarian surface epithelium (OSE) cytobrushing specimens was also obtained from the normal ovaries of patients at the time of surgery for benign indications. Frozen sections (7 µm) were affixed onto FRAME slides (Leica, Wetzlar, Germany), fixed in 70% alcohol for 30 seconds, stained by 1% methyl green, rinsed in deionized water, and air-dried. Microdissection was performed using a MD LMD laser microdissecting microscope (Leica). Tumor cells (∼5,000) were dissected for each case. RNA was isolated, extracted, and purified as previously described [15]. In order to generate sufficient cRNA for microarray analysis, a two-cycle amplification protocol (Affymetrix) was utilized that has been previously described [16].

### Microarray analysis

All array data is Minimum Information About a Microarray Experiment (MIAME) compliant and the raw data has been deposited in a MIAME compliant database (GEO, Accession Number: GSE29450)

#### Data Normalization

Global normalization at a target value of 500 was applied to all 20 of the arrays under consideration using Gene Chip Operating Software (Affymetrix). Normalized data were uploaded into the National Cancer Institute's Microarray Analysis Database (mAdb) for quality control screening and collation prior to downstream analyses (http://nciarray.nci.nih.gov/index.shtml). Biometric Research Branch (BRB) ArrayTools version 3.2.2 software developed by Drs. Richard Simon and Amy Peng Lam of the Biometrics Research Branch of the National Cancer Institute was used to filter and complete the statistical analysis of the array data. BRB-ArrayTools is a multifunctional Excel add-in that contains utilities for processing and analyzing microarray data using the R version 2.0.1 environment (R Development Core Team, 2004). Hybridization control probe sets and probe sets scored as absent at α1 = 0.05 or marginal (M) at α2 = 0.065 were excluded. In addition, only those transcripts present in greater than 50% of the arrays and displaying a variance in the top 50th percentile were evaluated.

#### Class comparison, gene ontology, and pathway analysis

A multivariate permutation test in BRB ArrayTools was utilized to identify differentially expressed genes. A list of probesets containing <10% false positives at a confidence of 95% was obtained after a total of 2,000 permutations. Differential expression was considered significant at a p-value of <0.001. A random variance *t* test and global assessment was performed as previously described [16].

In order to identify particular functional categories of genes that were highly enriched in CCOC, we identified gene ontology (GO) categories that were statistically significant among the list of differentially regulated genes. A Hotelling T-square test was performed to identify significant GO categories. In order to identify signaling pathways involved in CCOC, the gene list that was generated from the microarray analysis was imported into PathwayStudio software (Ariadne Inc, Rockville, MD).

### Quantitative RT-PCR

Quantitative real-time PCR was performed for the 10 clear cell ovarian cancer samples and the 10 OSE samples. 50 ng of the double-amplified product was used from all 20 samples using primer sets specific for 12 selected genes and the housekeeping genes GAPDH, GUSB, and cyclophillin. An iCycler iQ Real-Time PCR Detection System (Bio-Rad , Hercules, CA) was used in conjunction with the One-Step qRT-PCR with SYBR Green kit (Invitrogen Life Technologies, Inc., Carlsbad, CA) according to previously described cycling conditions [17]. To calculate the relative expression for each gene, the 2^−ΔΔCT^ method was used averaging the C_T_ values for the three housekeeping genes.

### Cell lines and culture conditions

The human clear cell ovarian cancer cell lines ES-2, TOV21G, and RMG1, and the serous cell lines OVCA 420 and OVCA 432 were maintained in a 1∶1 mixture of medium 199 (Invitrogen Life Technologies, Inc. Carlsbad, CA) and medium 105 (Sigma, St. Louis, MO), supplemented with 10% fetal bovine serum (FBS) and 1% L-glutamine. The human serous ovarian cancer cell lines SK-OV-3 and OVCAR-3 were maintained in RPMI 1640 (Invitrogen Life Technologies) supplemented with 10% FBS and 1% L-glutamine. CAOV3 and OVCA 429 cells were maintained in DMEM (Invitrogen Life Technologies) supplemented with 10% FBS and 1% L-glutamine. ES-2, TOV21G, SKOV3, OVCAR3 CAOV3 and RMG1 cell lines were purchased from American Type Culture Collection and Japanese Collection of Research Bioresources. OVCAR 420, OVCAR 432 and OVCA 429 were obtained from the Laboratory of Gynecologic Oncology at Brigham and Women's Hospital [18].

### Hypoxia/Glucose deprivation conditions

Cells from clear cell and serous ovarian origins were plated and placed in the conditions of normoxia with DMEM, 10% FBS,1% L-glutamine or hypoxia with glucose-free DMEM, 10% FBS,1% L-glutamine (Invitrogen Life Technologies). Hypoxia was produced by flushing an incubator (Thermo Forma Series II, Thermo Fischer Scientific, Inc., Waltham, MA) with nitrogen to achieve a mixture of 1% O_2_, 5% CO_2_, and 94% nitrogen. The incubator was maintained at 37°C.

### Cell proliferation assays

The cancer cell lines were seeded in 96-well plates in 8 replicates (ES-2, TOV21G, OVCA 420, SK-OV-3, OVCAR-3, OVCA-420, OVCA-429: 5×10^2^ cells per well, CAOV3, OVCA-432, RMG1: 1×10^3^ cells/well) and placed in either conditions of normoxia with media containing normal glucose (NN) or hypoxia/glucose deprivation (HG) for 24, 48, and 72 hours. After these periods, relative numbers of viable cells were measured using the fluorometric, resazurin-based Cell Titer Blue assay (Promega, Madison, WI) according to the manufacturer's instructions at 560_Ex_/590_Em_ nm in a Victor3 multi-label counter (PerkinElmer, Germany). Doubling times for each cell line under each condition were calculated using Prism 4.02 Software (Graph Pad, San Diego, USA). Fold change in doubling time of hypoxia/glucose deprivation compared to normal conditions was calculated and averaged for each cell line over two experiments. Average fold change was calculated for the serous cell lines and clear cell lines and compared.

### Cell cycling assay

The cell cycle status of the ES-2 and OVCA 420 cells were compared in the conditions of normoxia and hypoxia/glucose deprivation by flow cytometry. Briefly, 7.5×10^4^ ES2 cells and 2.0×10^5^ OVCA420 cells were seeded in 60 mm^2^ plates in triplicate and allowed to incubate overnight. The media was changed on the next day, and the cells were placed under the above conditions. After 48 hours of incubation (normoxia at 37°C or hypoxia/glucose deprivation at 37°C), the media and adherent cells were collected and centrifuged at 1500 χg for 5 minutes. The pellet was washed in 2 mls phosphate-buffered saline (PBS) and centrifuged at 1500 χg for 5 minutes. The cells were resuspended in 200 µl of cold PBS. 2 mls of ice-cold 70% ethanol was added, and the cells were incubated on ice for 30 minutes to permeabilize. The cells were then centrifuged at 1500 χg for 10 minutes. The supernatant was decanted and 900 µl of room temperature PBS was used to resuspend the cells. 100 µl of RNAse A (10 mg/ml, Worthington Biochemical Corp., Lakewood, NJ) and 10 µl of propidium iodide (1 mg/ml, Sigma) were added, and the tubes were then incubated at 37°C for 30 minutes, avoiding light. DNA contents were determined by flow cytometry (FACSCalibur, Becton, Dickinson, and Company, Franklin Lakes, NJ) and the histograms of DNA contents were analyzed using FlowJo 7.2 (Tree Star, Inc., Ashland, OR) to characterize the population fractions in each phase of the cell cycle.

### Caspase-3 assay

The direct measurements of caspase 3 activity were made using a caspase-3 fluorometric-kit (Invitrogen Life Technologies). Briefly, 2.0×10^5^ OVCA420 cells were seeded in 60 mm^2^ plates and allowed to incubate overnight. On Day 0, the media was replaced and the plates were then placed in the conditions of normoxia/normal glucose or hypoxia/glucose deprivation. Cells were collected at 24, 48, and 72 hours. The cells were collected, pelleted, resuspended in 50 µl of chilled Cell Lysis Buffer, and incubated on ice for 10 minutes. Protein concentration was determined by BCA protein assay (Pierce, Rockford, IL). 50 µl of 2× Reaction Buffer and 10 mM DTT were added to each 50 µl aliquot of cell lysate. 5 µl of 1 mM DEVD-AFC substrate was then added to each sample while avoiding the light. The samples were then incubated at 37°C for 1 hour in the dark. MEF cells (60 mm plate, 80% confluent) treated with 10 µl cycloheximide and 30 ng TNFα were used as a positive control. Fluorescence was then assessed in a Victor3 multi-label counter (PerkinElmer, Germany) with 405_Ex_/535_Em_ nm filters. The fold increase in Casase-3 activity was determined by relative fluorescence per µg protein.

### Necrosis assay

In order to assay for the presence of necrosis, the CytoTox-ONE™ homogeneous membrane integrity assay was used as recommended by the manufacturer (Promega). This assay measures the release of LDH from cells with damaged membranes. Briefly, 5×10^3^ OVCA-420 cells were plated in a 96 well plate in triplicate. The media was changed the next day, and the cells were placed in the conditions of either normal oxygen/normal glucose or hypoxia/glucose deprivation. 48 hours later, the plates were removed from the incubator and equilibrated to 22°C. In order to generate a Maximum LDH Release Control, 2 µl of lysis solution was added to the control wells placed in normal oxygen/normal glucose. 100 µl of CytoTox-ONE™ Reagent was added to each well. After 10 minutes of incubation at 22°C, 50 µl of Stop Solution was added to each well. The plates were shaken for 10 seconds. From each well, 100 µl was transferred to an opaque plate and fluorescence was recorded at 560_Ex_/590_Em_ nm in a Victor3 multi-label counter (PerkinElmer, Germany). After subtracting the culture medium background, the percent cytotoxicity was calculated by the dividing the experimental fluorescence by the Maximum LDH Release fluorescence.

### Treatment of cell lines with siRNA oligonucleotides

Knockdown of HIF1 α and Enolase 1 was performed using siRNA oligonucleotides (Qiagen, Inc). A reverse transfection protocol was performed using Oligofectamine (Invitrogen Life Technologies, Inc) as recommended by the manufacturer in a 96-well plate format. For each well, 50 nM of siRNA and 0.5 µl Oligofectamine transfection reagent was diluted in 50 µl of serum-free DMEM and allowed to incubate at room temperature for 30 minutes. ES-2 cells, TOV-21G cells, and RMG-1 cells were seeded in a 96-well plate at 1.0×10^4^ cells, 2.0×10^4^ cells, and 5.0×10^4^ cells per well, respectively. Scrambled siRNA was used as a negative control. 48 hours after transfection, the media was replaced with glucose-free DMEM, and cells were placed under the conditions of hypoxia. Growth was assessed at 0 and 24 hours by Cell Titer Blue assay (Promega). The proliferation assays for ES2 and TOV21G cell lines were performed using 75 nM siRNA for 24, 48 and 72 hours after transfection.

### *In vivo* reduction at ENO1 or HIF1 α using siRNA-DOPC with or without sunitinib inhibits CCOC tumor progression in athymic nude mice

Female athymic nude mice were purchased from the National Cancer Institute, Frederick Cancer Research and Development Center (Frederick, MD). ES2 cells were trypsinized, washed and resuspended in Hanks' balanced salt solution (Gibco, Carlsbad, CA) and injected intraperitoneal into mice (1×10^6^ cells/mouse). In order to down-regulate Enolase and HIF1 α in vivo, the respective siRNA was employed. Non-targeting, nonspecific sequence 5′-ATT TCT CCG AAC GTG TCA CGT-3′ was used as control, while the human-specific sequences were used for Enolase (5′-GCG CAT TGG AGC AGC AGA GGT TTA3′) and HIF-1 α (5′ CAG TTG TCA CCA TTA GAA A-3′). These target specific siRNAs were purchased from Sigma-Aldrich and prepared as previously described [19], [20]. The lyophilized DOPC incorporated siRNA was hydrated with PBS and injected intraperitoneal twice weekly following our previously published protocols [21] at 5.0 mg siRNA/200 mL suspension. The same volume of PBS injected intraperitoneal was used as a control.

SU11248/sunitinib malate (Sutent, Pfizer) was suspended in carboxymethylcellulose vehicle formulation, containing carboxymethylcellulose sodium (0.5% wt/vol), NaCl (1.8% wt/vol), Tween 80 (0.4% wt/vol), benzyl alcohol (0.9% wt/vol), and reverse osmosis deionized water (added to final volume) and adjusted to pH 6.0 as previously described [22]. Sunitinib structure and activity have previously been reported [23]. Drug aliquots were prepared once weekly and kept in the dark at 4°C. Mice were treated with 40 mg/kg in 200 µl vehicle by gavage once daily. Daily gavage with vehicle alone was used as a control. Seven days after ES2 cell injection, mice were randomly divided and treated with control, Enolase or HIF1 α siRNA-DOPC ± sunitinib (n = 10/group). Treatment was continued for 3 weeks, at which point, all mice in the experiment were sacrificed and necropsied, and tumors were harvested. Tumor weight and nodule count were recorded. Tumor tissue was frozen in optimal cutting temperature (OCT) media to prepare frozen slides for subsequent CD31 staining.

### Immunohistochemical staining for CD31

Immunohistochemical staining for CD31 antigen was performed on frozen slides to evaluate tumor microvessel density (MVD). Slides were fixed in cold acetone for 10 minutes. Endogenous peroxidase was blocked with 3% H_2_O_2_ in methanol and nonspecific epitopes were blocked using 5% normal horse serum and 1% normal goat serum. Slides were then incubated with anti-mouse CD31 (1∶800 dilution, PharMingen San Diego, CA) at 4 degrees overnight. After washing with PBS, the appropriate HRP-conjugated secondary antibody in blocking solution was added for 1 hour at room temperature. Slides were developed with 3, 3″-diaminobenzidine (DAB) chromogen (Invitrogen, Carlsbad, CA) and counterstained with Gil No. 3 hematoxylin (Sigma-Aldrich, St. Louis, MO). MVD was calculated by viewing 10 representative 200× fields per slide in each treatment group and counting the number of microvessels per field. A microvessel was defined as an open lumen with at least one CD31-positive cell immediately adjacent to it.

### Differential effect of sunitinib on transplantable patient-derived ovarian cancer tumors

Six- to eight week old female NOD-SCID mice were bred by the BC Cancer Research Centre Animal Resource Centre, BC Cancer Agency, Vancouver, Canada. Mice were housed under specific, pathogen-free conditions in sterile filter-top cages in high efficiency particulate air-filtered ventilated racks, and received sterile rodent chow and water. Ovarian cancer tissue specimens were obtained with informed consent from patients undergoing bilateral salpingoophorectomy at Vancouver General Hospital. Briefly, to develop transplantable cancer tissue lines, fresh tumor tissue was cut into small pieces and grafted into the subrenal capsule site of female NOD-SCID mice for subsequent serial transplantation and characterization as previously described [24], [25]. A panel of ovarian cancer tissue xenograft models, i.e., three serous carcinoma tissue lines (LTL237, 247 and 259) and one clear cell carcinoma tissue line (LTL175) (http://livingtumorlab.com/PDC_Ovarian.html), was used.

For Sunitinib efficacy studies, 96 pieces of tissue (4×2×1 mm^3^) from xenografts of each established tumor tissue line were grafted under renal capsules of 24 female NOD-SCID mice, as previously described [25]. When the implants were well-established, reaching an average volume of about 20 to 50 mm^3^ [24], [25], the animals were sorted into four groups (6 mice/group; 2 grafts per kidney). Treatment assignments were to sunitinib or inactive vehicle (negative control). Sunitinib was administered as a 0.5% carboxymethyl cellulose suspension using a dosage of 40 mg/kg body weight (orally; once daily, for two weeks) found efficacious for a variety of mouse xenograft models, as described elsewhere [26]. The mice were provided with food and water *ad libitum* and monitored daily for changes in general health and signs of stress, including body weight loss, diarrhea, changes in food/water intake, appearance (hunched posture, sunken eye, labored breathing) and behavior (lethargy). Effects on tumor growth were assessed by measurement of tumor volume at necropsy using calipers and the formula: volume (mm^3^) = 0.52×length×width×height (in mm), as previously described [25] and by histochemical analysis of tumor tissue sections (see below).

### Measurement of tyrosine phosphorylation of VEGFR2 and PDGFRβ in xenografts via Western blotting

Within 2 h of the last administration of sunitinib in the above efficacy studies, xenografts from treated and control mice were snap frozen in liquid nitrogen and stored at −80 C for subsequent use. Lysates were prepared by homogenization of tissues with cold lysis buffer (50 mM HEPES pH 7.5, 150 mM NaCl, 1.5 nM MgCl_2_, 10% v/v glycerol, 1% Triton X-100, 1% sodium orthovanadate, 2 mM NaF, 2 µg/mL aprotinin, 2 µg/mL leupeptin, and 2 µg/mL pepstatin-A) as described elsewhere [26]. For immunoprecipitation, protein amounts were adjusted to 500 µg. Proteins were precleaned with protein A-Sepharose (Cat. No. 16-125, Upstate Biotechnology Inc., Lake Placid, NY) for 15 min at 4C. Supernatants (1 mL) were incubated with 4 µg rabbit anti-VEGFR2 (Cat. No. 07-716) or anti-PDGFRβ (Cat. No. 05-825) antibodies (Upstate Biotechnology Inc.) for 90 min at 4C and, following addition of 25 µL 50% (v/v) Protein A-Sepharose, further incubated for 60 min at 4C. Antigen-antibody-bead complexes were washed at least three times with lysis buffer, followed by addition of 25 µL loading buffer and boiling for 1 min for protein elution. Proteins were separated by 5% SDS-PAGE gel and then transferred onto PVDF membrane for detection of phosphorylated tyrosine using a mouse monoclonal antibody to phosphotyrosine (Cat. No. sc-7020, Santa Cruz Biotechnology Inc.). Membranes were subsequently stripped and reprobed for detection of total VEGFR2 and PDGFRβ using the same antibody preparations used for immunoprecipitation.

### Apoptosis detection

Paraffin-embedded tissue sections (5 µm thick) were examined by TUNEL assay (ApopTag® Apoptosis Detection Kit, Chemicon, Temecula, CA) as previously described (26). Briefly, sections were incubated for 15 min with 20 µg/mL proteinase K at room temperature and then thoroughly washed in distilled water. DNA fragments produced by the apoptotic process were tagged with digoxigenin nucleotides via a 60 min incubation at 37°C with terminal deoxynucleotidyl transferase (TdT) in a humidity chamber. The sections were then rinsed and incubated for 30 min at room temperature with anti-digoxigenin conjugated with fluorescein. After counterstaining with 4′-6-diamidino-2-phenylindole (DAPI), the slides were examined for the percentage of fluorescein-tagged cells using a Zeiss Axioplan-2 fluorescence microscope.

### Histology, immunohistochemistry, and microvessel density estimation

Preparation of paraffin-embedded tissue sections, their staining and immunohistochemical analyses were carried out as previously described (23, 24). Anti-von Willebrand factor VIII antibody (Cat. No. A0082, DAKO Diagnostics Canada Inc.; 1∶200) was used for identification of microvessels. All tissue sections were lightly counterstained with 5% (w/v) Harris hematoxylin (H&E). Control sections were processed in parallel with rabbit non-immune IgG (Dako, Carpinteria, CA) used at the same concentrations as the primary antibodies. Microvessel density, i.e., the number of blood vessels per ×400 microscopic field, was determined via microscopic analysis of von Willebrand factor VIII-stained tissue sections.

### Statistical analysis

Data are expressed as means ± S.E.M. ANOVA was used to compare multiple group mean values. Shaffer statistics and Student's t-test were used for analyzing any difference between two groups. Comparison of the percentage of cells in different cell cycle phases was performed with two-way ANOVA with use of Prism 4.02 Software (Graph Pad). Values were considered statistically significant at P<0.05, except for the microarray analysis, where significance was set at p<0.001.

## Results

### Whole genome expression profiles of microdissected CCOC identifies differentially expressed genes

**Figure 1:**
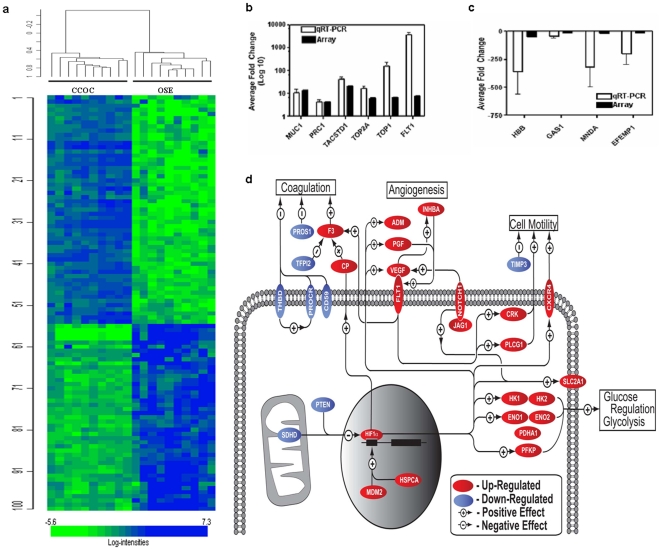
Whole genome expression profiling of clear cell ovarian tumors. (**a**) Graphic representation of whole genome expression profiling of the Clear Cell Ovarian Cancer Specimen (CCOC) and Ovarian Surface Epithelium (OSE). (**b**) and (**c**) Comparison of qRT-PCR data and microarray data of the six overexpressed and four underexpressed genes used for validation (**d**) Pathway analysis of differentially regulated genes identified in the clear cell ovarian cancer microarray. Genes included in the analysis were required to have a fold change ≥1.5. Multiple probe sets were averaged for each gene. *Red*, gene is up-regulated. *Blue*, gene is down-regulated.

The gene expression patterns of RNA isolated from the epithelial component of 10 clear cell ovarian cancer specimens isolated by laser capture microdissection were compared to similarly isolated RNA from 10 normal ovarian surface epithelium specimens using Affymetrix U133 plus 2 arrays. After normalization and initial analysis, 16,013 informative probesets passed filtering criteria. Using a multivariate permutation test providing 95% confidence that the number of false discoveries did not exceed 10%, 3,288 probesets for 2,559 genes were found to be differentially regulated, defined by a 1.5-fold or greater difference in expression with a statistical significance of p<0.001. Graphic representation of this differential gene expression can be seen in Figure 1a. To validate the microarray data, 12 genes that were differentially expressed were randomly selected for qRT-PCR analysis. Ten of the 12 genes were differentially expressed on qRT-PCR, giving an overall microarray validation rate of 83% (Figure 1b, c and Table S1).

### Identification of activated pathways in CCOC

In order to identify activated pathways present within CCOC, functional categories of differentially expressed genes were identified using gene ontology (GO) analysis. Statistically significant categories demonstrate a large number of genes involved in carbohydrate metabolism, glucose metabolism, glycolysis, and blood vessel development (Table 1). The 3,288 probesets and their associated relative expression data when compared to OSE were imported into PathwayStudio 6.0 software. Pathways involved in angiogenesis, coagulation, glucose metabolism, cell proliferation, and cell motility were evident (Figure 1d). For instance, HSPCA, which has been shown to stabilize HIF1α (27), was found to be over-expressed. Genes regulated by HIF1α involving glycolysis (*ENOl* [27] and *SLC2A1* [28]) and angiogenesis (*PGF* [29], *VEGF* [30], and *FLT1* [31]) were identified. Both *PROS1* and *F3* were found to be dysregulated. *F3* is up-regulated by FLT1 [32] and CP [33]. NOTCH1, which was found to be over-expressed, has been shown to up-regulate both *VEGF* [34] and *SLC2A1* [35], contributing to both the angiogenesis and glycolysis pathways. Table 2 provides a more detailed description of these genes.

**Table 1:** Gene Ontology categories found to have a statistically significant higher number of genes than expected by chance.

GO Category	No. genes	p value
Cytoskeleton	76	p<1e-07
Cell cycle	39	p<1e-07
DNA metabolism	37	p<1e-07
Carbohydrate metabolism	27	p<1e-07
Cell motility	17	p<1e-07
Blood coagulation	12	p<1e-07
Glucose metabolism	10	p<1e-07
Cell growth	10	p<1e-07
Glycolysis	8	p<1e-07
DNA repair	8	p<1e-07
Blood vessel development	5	p<1e-07
Microtubule cytoskeleton organization and biogenesis	5	p<1e-07

**Table 2:** Pathway genes.

Entrez gene ID	Gene	Description	Fold change	Function
133	*ADM*	Adrenomedullin	4.37	Angiogenesis, Cell proliferation and invasion.
966	*CD59*	CD59, Complement regulatory protein	−2.47	Inhibitor of complement membrane attack complex (MAC) action
1356	*CP*	Ceruloplasmin	13.78	Copper homeostasis
1398	*CRK*	v-crk sarcoma virus CT10 oncogene homolog (avian)	1.86	Cell proliferation, focal adhesion Cell motility
7852	*CXCR4*	Chemokine (C-X-C motif) receptor 4	4.42	Cell invasion and motility
2023	*ENO1*	Enolase 1 (alpha)	2.21	Glycolysis
2026	*ENO2*	Enolase 2(gamma, neuronal)	2.39	Glycolysis
2152	*F3*	Coagulation factor III (thromboplastin, tissue factor)	4.71	Thrombosis
2321	*FLT1*	fms-related tyrosine kinase 1	5.63	VEGF receptor, angiogenesis
3091	*HIF1A*	Hypoxia-inducible factor 1, alpha subunit	2.64	Promoter for genes involved in angiogenesis and glycolysis.
3098	*HK1*	Hexokinase 1	4.18	Glycolysis
3099	*HK2*	Hexokinase 2	5.85	Glycolysis
3320	*HSPCA*	Heat shock protein 90kDa alpha, class A member 1	2.874	Protein binding
3624	*INHBA*	Inhibin, beta A	4.16	Angiogenesis
182	*JAG1*	Jagged 1 (Alagille syndrome)	2.61	Notch ligand, cell proliferation
4193	*MDM2*	Mdm2, transformed 3T3 cell double minute 2	2.92	Oncogene
4851	*NOTCH1*	Notch homolog 1, translocation-associated (Drosphilia)	8.6	Cell fate decisions
5160	*PDHA1*	Pyruvate dehydrogenase (lipoamide) alpha 1	2.09	Glycolysis
5214	*PFKP*	Phosphofructokinase, platelet	4.3	Glycolysis
5228	*PGF*	Placental growth factor	3.35	Angiogenesis
5335	*PLCG1*	Phospholipase C, gamma 1	3.07	Cell motility
10544	*PROCR*	Protein C receptor, endothelial (EPCR)	−14.93	Binds activated protein C, inhibiting blood coagulation
5627	*PROS1*	Protein S (alpha)	−5.1	Prevents coagulation and stimulates fibrinolysis
5728	*PTEN*	Phosphatase and tensin homolog (mutated in multiple advanced cancers 1)	−2.43	Tumor suppressor
6392	*SDHD*	Succinate dehydrogenase complex, subunit D, integral membrane protein	−1.82	HIF1α degradation
6513	*SLC2A1*	Solute carrier family 2 (facilitated glucose transporter), member 1	3.76	Glucose transport
7980	*TFPI2*	Tissue factor pathway inhibitor 2	−2.47	Inhibits tissue factor
7056	*THBD*	Thrombomodulin	−4.44	Activates protein C, inhibiting blood coagulation
7078	*TIMP3*	TIMP metallopeptidase 3 (Sorsby fundus dystrophy, pseudoinflammatory)	−5.35	Inhibitor of matrix metalloproteinases
7422	*VEGF*	Vascular endothelial growth factor	2.71	Angiogenesis

Differentially expressed genes identified in the clear cell microarray involved in coagulation, angiogenesis, cell proliferation, cell motility, and glucose metabolism (average fold change ≥1.5; P<0.001).

### CCOC are resistant to hypoxia/glucose deprivation induced necrotic death

**Figure 2:**
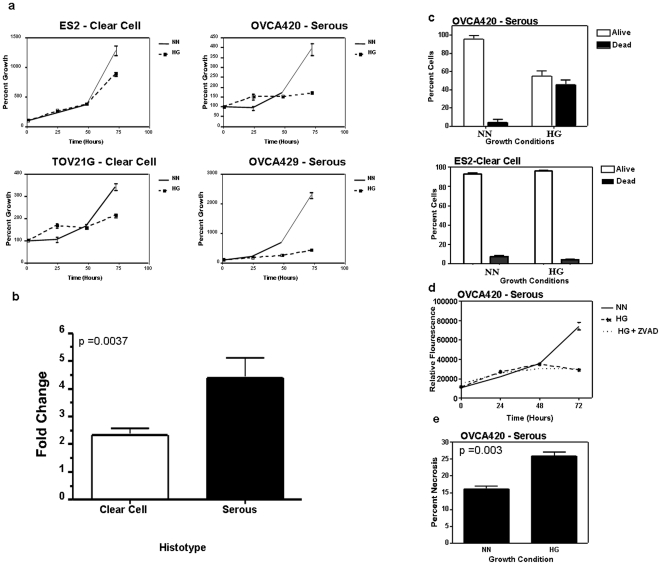
Clear cell ovarian cancer cell lines were more resistant to hypoxia/glucose deprivation than serous ovarian cancer cell lines. (**a**) Cellular proliferation assays of ovarian cancer cell lines of clear cell and serous origin under different oxygen and glucose conditions. The doubling times of the cell lines were compared, and the fold change in doubling time between the conditions of normal oxygen/normal glucose (NN) and hypoxia/glucose deprivation (HG) were calculated. (**b**) The bar graph demonstrates a statistically significant difference when the fold change in doubling times is averaged by histotype, showing that the three clear cell ovarian cancer cell lines were less affected by hypoxia/glucose deprivation than the six serous cell lines (p = 0.0037). (**c**)Trypan blue exclusion assay of OVCA-420 and ES2 cells grown in normal oxygen/normal glucose (NN) and hypoxia/ glucose deprivation (HG) for 72 hours. (**d**) Proliferation assay of OVCA-420 cells incubated in NN, HG, and HG with Z-VAD-FAK. The addition of Z-VAD-FAK did not alter the growth inhibition of HG. (**e**) Necrosis assay of OVCA-420 cells demonstrated a statistically significant increase in necrosis when incubated in HG (p = 0.003).

The presence of a dominant activated pathway involving angiogenesis and glycolysis in ovarian clear cell tumors suggested that these cells have developed mechanisms to survive in low oxygen and glucose conditions. This provides survival signals for clear cell tumors under conditions where other tumors might die. We tested the growth of ovarian cancer cell lines of papillary serous and clear cell origin under the conditions of normoxia/normal glucose (NN) compared with to hypoxia (1% O2) and glucose deprivation (HG). Deprivation of oxygen and glucose had a minimal effect on the growth of clear cell cell lines (ES2 and TOV21G) when compared to serous cell lines (OVCAR 420 and OVCAR429) which were essentially completely growth inhibited (Figure 2a). Further we compared the doubling times of the cell lines and the fold change in doubling time between the conditions of NN and HG were calculated. The bar graph demonstrates a statistically significant difference when the fold change in doubling times is averaged by histotype, showing that the three clear cell ovarian cancer cell lines were less affected by hypoxia/glucose deprivation than the six serous cell lines (Figure 2b. p = 0.0037). These results highlight the importance of hypoxia-related and glycolysis pathways in clear cell ovarian cancer.

A cell cycle analysis of serous cell lines after 48 hours in the conditions of HG demonstrated a significant increase in the G1 phase as well as a decrease in S phase under the HG conditions. However, only OVCA-420 demonstrated a more global effect of HG with a significant increase in both G2/M phase (Figure S1). In addition to cell cycle arrest, we observed a large portion of OVCA-420 cells were detached after 48–72 hours of HG conditions, which was not seen in ES-2 clear cell cell lines. Trypan blue exclusion assay was performed on both cell lines which confirmed that 40% of cells under HG were dead for OVCAR-420 and ES2 cell lines were resistant to HG conditions (Figure 2c). To determine the mechanism of cellular death, a proliferation assay was performed under treatment with Z-VAD-FAK to see if cell death was prevented. No decrease in cell death was noted suggesting that apoptosis was not a major contributor to cellular death (Figure 2d). This was supported by the lack of caspase activity after 24, 48, and 72 hours (Figure S2). A cellular necrosis assay was performed on OVCA-420 cells grown in hypoxia/glucose deprivation and showed a 100% increase in necrosis (Figure 2e).

### siRNA targeting *HIF1* α or *ENO1* sensitizes CCOC cell lines to HG conditions

**Figure 3:**
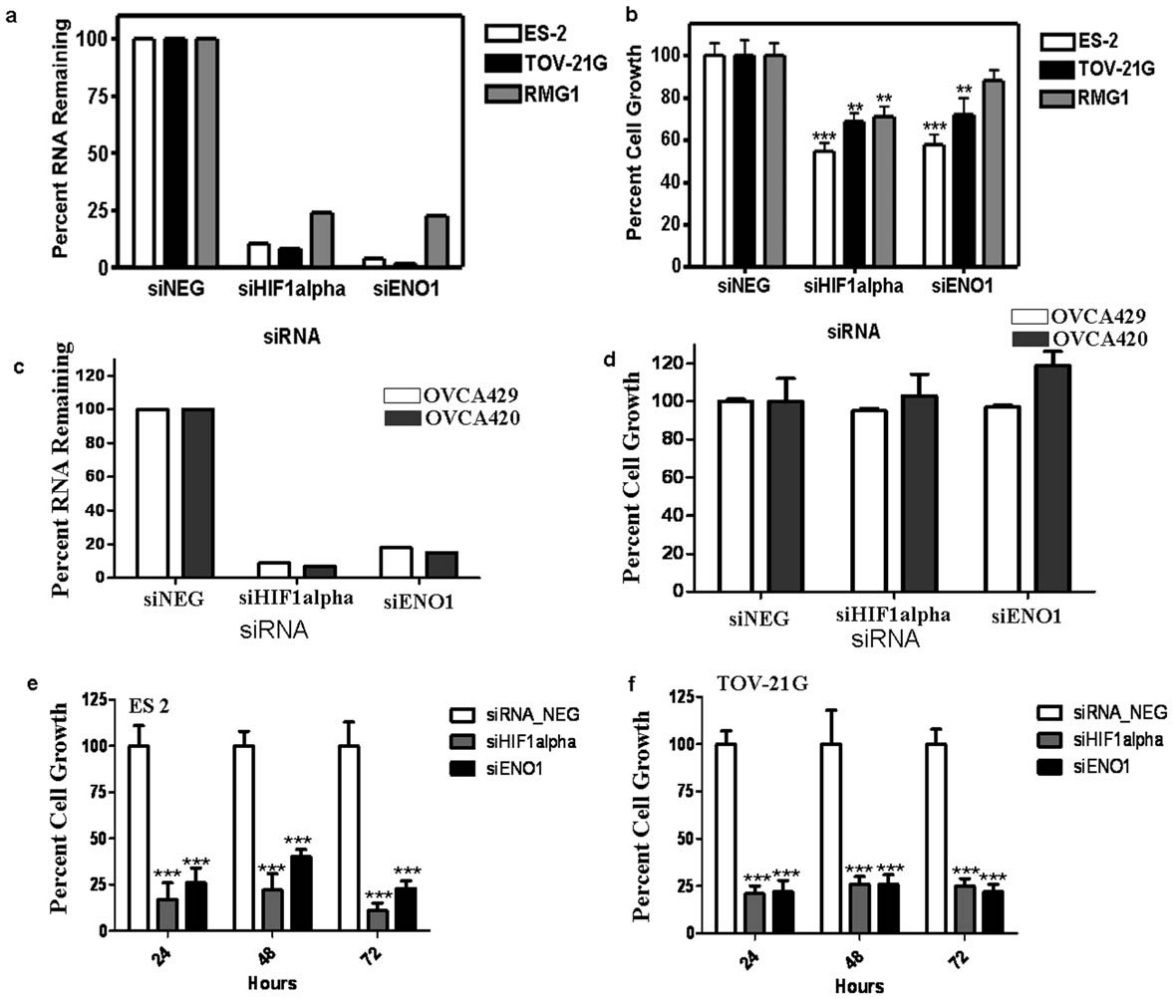
Knockdown of HIF1α and ENO1 in three clear cell ovarian cancer cell lines. (**a**) Knockdown efficiency of siRNA molecules targeting HIF1α and ENO1 in three clear cell ovarian cancer cell lines, assessed by quantitative real-time PCR. (**b**) The percent of growth inhibition after transfection of siRNA molecules targeting HIF1α and ENO1. Growth was assessed after 24 hours of hypoxia/glucose deprivation *p = 0.12, **p<0.05,***p = <0.0001. (c, d) Knockdown efficiency of siRNA molecules targeting HIF1α and ENO1 in OVCA429 and OVCA420 and the percent of growth inhibition after transfection of siRNA molecules targeting HIF1α and ENO1. (e, f) Effect of HIF1α and ENO1 knockdown on proliferation of ES-2 and TOV-21G clear cell cell lines (***p = <0.0001).

The microarray results and the *in vitro* HG assays suggested that clear cell cancers were protected from the growth inhibiting effects of these stresses by the activation of specific pathways. To validate this mechanistically we “knocked-down” key genes in these pathways by using siRNA technology. Three clear cell cancer lines (ES-2, TOV21G, and RMG1) were transfected with siRNA targeting *HIF1* α (hypoxia) and *ENO1* (glycolysis). Reduction of the expression of both genes at these cell lines (Figure 3a) demonstrated significant growth inhibition to HG conditions compared to control transfected cells. Both ES-2 and TOV21G cells demonstrated statistically significant growth inhibition when transfected with siRNA targeting *ENO1* (Figure 3b). The degree of knockdown efficiency of ENO1 correlated with the amount of growth inhibition; while RMG1 demonstrated growth inhibition with knockdown of ENO1, it was the least efficient of the three clear cell cell lines and this amount of growth inhibition was not statistically significant (p = 0.12). Serous ovarian cancer cell lines OVCA420 and OVCA429 were growth inhibited in hypoxia (1% O2) and glucose deprivation (HG) conditions and the transfection with siRNA targeting *HIF1 α* and *ENO1* has limited effect on further growth inhibition compared to the control transfected cells (Figure 3c and d). Further the Knockdown of HIF1 α and Enolase 1 was performed on clear cell cell lines ES-2 and TOV21G using siRNA. Forty eight hours after transfection, the proliferation assays were carried out for hypoxia/glucose deprivation for another 24, 48, and 72 hour. This experiment supports that HIF1 α and Enolase 1 expression are important for the ability of clear cell ovarian cells to grow efficiently in hypoxic/low glucose conditions (Figure 3e and f).

### *In vivo* reduction at ENO1 or HIF1 α using siRNA-DOPC with or without sunitinib inhibits CCOC tumor progression in athymic nude mice

**Figure 4:**
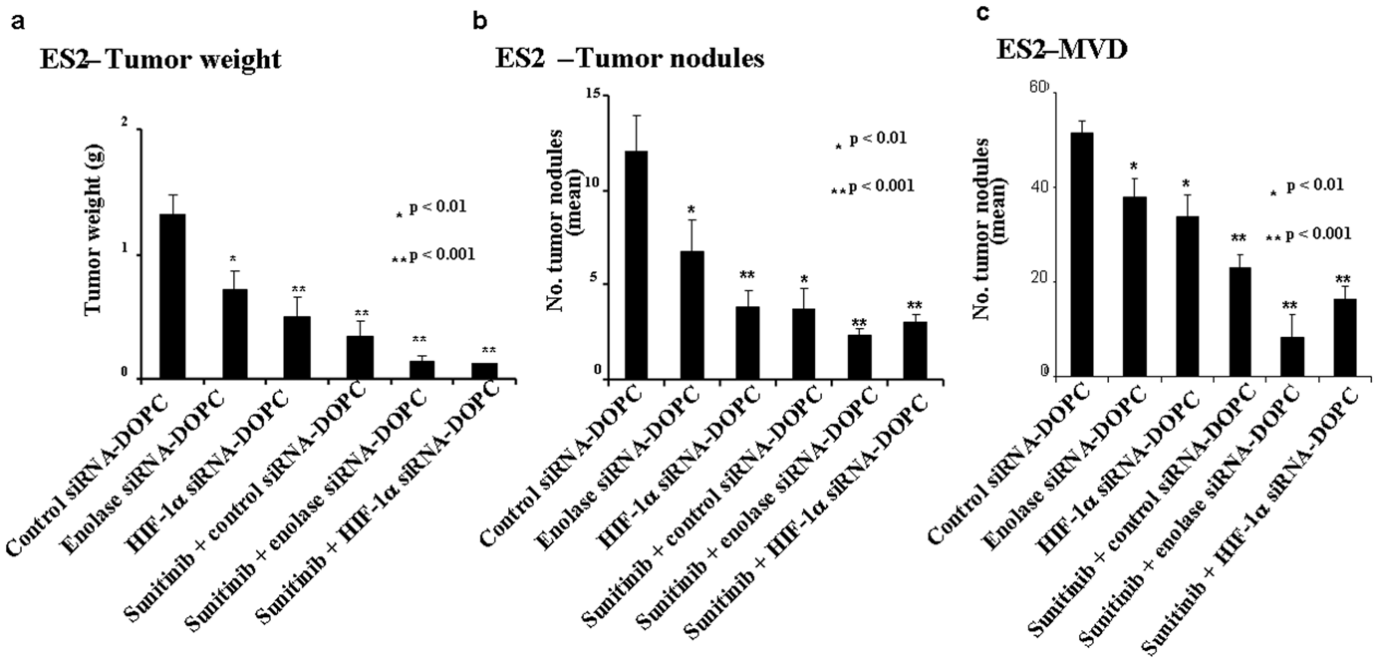
Effect of Enolase or HIF1 α siRNA-DOPC ± sunitinib on ovarian clear cell tumor progression in the female athymic nude mouse model. (**a**) Tumor weight: ES2 cells (1×10^6^ cells/mouse) were injected intraperitoneal into mice. The mice were treated with control, Enolase or HIF1 α siRNA-DOPC ± sunitinib (n = 10/group). Immunohistochemical staining for CD31 antigen was performed on frozen slides of tumor to evaluate the number of tumor nodules (**b**) and tumor microvessel density (MVD) (**c**). (See Materials and Methods section for details).

As our microarray pathway analysis revealed angiogenesis as a critical pathway in clear cell ovarian cancer growth, we hypothesize that inhibition of angiogenesis should have potent effects on clear cell ovarian cancer growth. To test this, we evaluated the therapeutic potential of sunitinib in clear cell ovarian cancer. Sunitinib is a small molecule inhibitor of receptor tyrosine kinases (TKIs) such as VEGFR (vascular endothelial growth factor receptor) and PDGFR (platelet-derived growth factor receptor) tyrosine kinases. While we could have used other TKIs, sunitinib was easily available and in preclinical studies. It was found to exhibit robust antitumor and antiangiogenic activity in a variety of cancer models. Inhibition by sunitinib of VEGF and PDGF signaling pathways appears to be particularly critical for tumor-induced angiogenesis. Recently, sunitinib has been approved by the FDA for clinical therapy of renal clear cell carcinoma and gastro-intestinal stromal tumors. To functionally validate the pathways *in vivo*, we applied siRNA technology along with antiangiogenic agents in athymic nude mice orthotopic models. The ES2 cell line was used since it reproducibly produced large tumors over a short period of time. Tumor weight was found to be significantly reduced in *ENO1* and *HIF1α* siRNA treated mice with respect to its control group (Figure 4a). Likewise treatment of the mice with antiangiogenic agent (sunitinib) produced large reduction in tumor weight. Combination of siRNA and antiangiogenic agents produced synergistic activity. A similar trend was observed for tumor nodule and tumor microvessel density estimations (Figure 4b,c).

### Growth of patient-derived CCOC tissue xenografts sensitive to sunitinib

**Figure 5:**
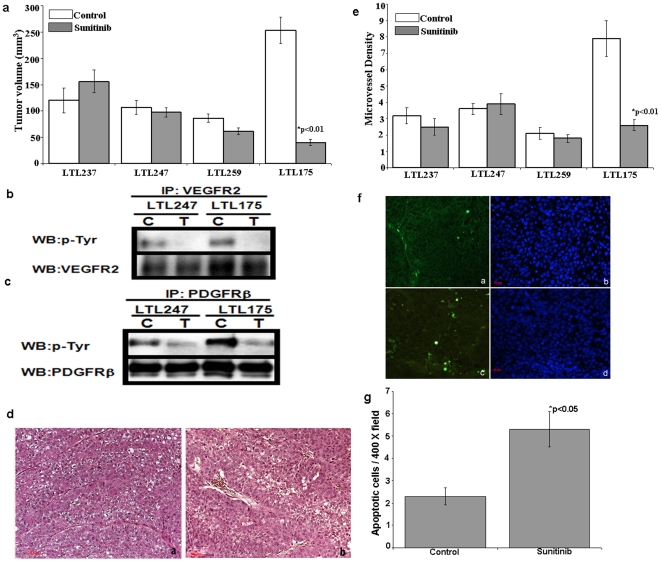
Effect of sunitinib on the growth of patient-derived CCOC tissue xenografts. (**a.**) Effect of a two-week treatment with sunitinib on growth of subrenal capsule xenografts in NOD-SCID mice (6 mice/group; 2 grafts per kidney) of transplantable serous (LTL237, 247 and 259) and clear cell (LTL175) ovarian carcinoma tissue lines derived from patients' cancers. Growth of the xenografts is expressed as % tumor volume determined at necropsy by measurement with calipers. Data are presented as means ± S.E.M. (**b, c**) Effect of sunitinib on VEGFR2 and PDGFRβ tyrosine phosphorylation as shown by Western blot analysis. Subrenal capsule xenografts of ovarian LTL247 serous carcinoma tissue and LTL175 clear cell carcinoma tissue in mice, treated for 2 weeks with sunitinib or vehicle (control), were lysed and processed for Western blot analysis of VEGFR2 (**b**) and PDGFRβ (**c**) tyrosine residues. The results are representative of 3 experiments. (**d**) Representative H&E-stained tumor sections of control (**d.a**), and sunitinib-treated (**d.b**) LTL175 tissue. (**e**) Effect of sunitinib on microvessel density of subrenal capsule xenografts in NOD-SCID mice of serous (LTL237, 247, 259) and clear cell (LTL175) ovarian carcinoma tissue lines. Data are presented as the average number of blood vessels per ×400 microscopic field ± S.E.M. (**f**) Effect of sunitinib on apoptosis in LTL175 xenografts in NOD-SCID mice treated with sunitinib for 2 weeks. Representative tissue sections of control (**f. a**) and sunitinib-treated (**f. c**) TUNEL-stained tumor tissue and counterstained with DAPI (**f. b**, control; **f. d**, sunitinib-treated); (**g**), percentage of apoptotic cells determined via microscopic analysis using a 400× microscopic field; data presented as means ± S.E.M.

As cancer models based on xenografts of cultured cancer cell lines in general do not adequately represent the disease as it present in humans, we used patient derived clear cell and serous ovarian cancer tumors grafted under the renal capsule of NOD-SCID mice to mimic the clinically relevant cancers. This method allows high tissue perfusion and potentially rapid development of graft microvasculature. More importantly, the ovarian cancer tissues can be grown and serially transplanted under renal capsules of NOD-SCID mice, with minimal histological and genetic changes, and with retention of sensitivity to cytotoxic chemotherapy. These patient-derived cancer tissue lines are therefore very similar to the original cancer specimens and, as such, their xenografts in NOD-SCID mice provide ovarian cancer models that closely resemble the patients' malignancies. To further validate our findings we tested patient tumor explants directly in NOD-SCID mice. As shown in Figure 5a, treatment with sunitinib markedly inhibited growth of the LTL175 clear cell cancer (P<0.01), whereas it had essentially no effect on the growth of the serous carcinoma lines LTL237, 247 and 259 (stable tumor size). To confirm that the sunitinib had receptor tyrosine kinase inhibitory activity *in vivo*, lysates of LTL247 and LTL175 xenografts were treated for 2 weeks with sunitinib or vehicle (control) and were examined by western blotting for amounts of phosphorylated tyrosine residues on VEGFR2 and PDGFRβ proteins. As shown in Figure 5b and 5c, there was active tyrosine phosphorylation of both VEGFR2 and PDGFRβ in the two tumors. The 2-week treatment with sunitinib reduced tyrosine phosphorylation of VEGFR2 and PDGFRβ in both the serous carcinoma (LTL247) and CCOC (LTL175) tissues to very low levels. Our genomic data supports a model where clear cell cancers of the ovary have activated pathways involving angiogenesis. We propose that this explains, in part, their clinical aggressive behavior and rapid tumor growth. As such, we hypothesize their growth would be exquisitely sensitive to antiangiogenesis agents. Serous tumors appear to have minimal activation of this pathway and as such should be somewhat insensitive to these agents. The result of this experiment demonstrates that there is sufficient sunitinib to inhibit the target but the serous tumors are less dependent on these pathways than clear cell tumors. This experiment confirms this but it is critical to ensure this is not the result of lack of drug effect on its target.

### Differential effect of sunitinib treatment on tumor mass and cell morphology/viability of CCOC xenografts correlate with microvessel density

Subrenal capsule LTL175 xenografts treated for two weeks with vehicle (controls) showed markedly enlarged tumor masses (Figure 5a) which, as indicated by H&E staining of tissue sections, consisted of viable cancer cells (Figure 5d.a). In contrast, sunitinib-treated tumors showed much lower tumor mass and lower cell numbers (Figure 5d.b) than controls. Since sunitinib has anti-angiogenic activity [26], we investigated the effect of the 2-week treatment with sunitinib on the microvessel densities of LTL237, 247, 259 and 175 tumors by microscopic analysis of von Willebrand factor VIII-stained tumor sections. Whereas sunitinib had no significant effect on the microvessel densities of the tumors of the three serous carcinoma tissue lines, it led to markedly lower (∼66%; P<0.01) microvessel density of the LTL175 clear cell carcinoma tumors (Figure 5e).

### Effect of sunitinib on apoptosis of LTL175 tumors

Immunohistochemical analysis of TUNEL-stained tissue sections showed that LTL175 ovarian clear cell carcinoma xenografts, treated for 2 weeks with sunitinib, had a substantially higher number of apoptotic cells than control xenografts (Figure 5f). On average, the number of apoptotic cells in a 400× microscopic field was approximately 2-fold higher in the sunitinib-treated tumor tissue (P<0.05) (Figure 5g).

## Discussion

This is the first report of whole genome expression profiling of microdissected CCOC specimens. We carried out a direct comparison of our gene list with a publically available datasets of clinical samples (GSE6008) [36]. GSE6008 contains expression profiling for 8 clear cell carcinomas and 4 individual normal ovary samples using Affymetrix HG_U133A array. Robust Mult-chip Average (RMA) analysis identified 3365 genes using a two sample T-test (p<0.001). Comparison of this gene list with our CCOC gene list identified 731 common genes. Pearson's Chi-squared test with Yates' continuity correction identified the overlap between the two sets of genes (731 genes) is significant and not due to chance (p-value<0.001). These 731 genes correspond to 29% of our CCOC gene list. We were not expecting a large overlap in these datasets because we generated our CCOC gene list from the laser capture microdissected epithelial component of clear cell ovarian cancers. These are pure populations of epithelial cells without the contamination of stromal components. In another sudy, Yamaguchi et al derived an ovarian clear cell carcinoma gene signature using cell lines and clinical samples expression analysis [37]. They have identified a 437 probe sets corresponding to 320 genes as the CCOC signature. We have found 76 genes from this 320 genes overlap to our gene signature and which was highly significant (p-value<0.001). Of note, the CCOC signature reported by Yamaguchi includes molecular networks of hypoxia-inducible factor 1 (HIF1α) which is reported in our study.

Comparison of the CCOC microarray gene list with a similarly generated gene list from microdissected serous ovarian tumors [38], identified that 73% of genes in the list were unique to CCOC. These unique genes likely explain the clinico-pathologic properties of CCOC. For instance, a large number of genes involved in coagulation were also found to be dysregulated, which may explain why patients with CCOC have been found to have a higher incidence of thromboembolic events when compared to patients with other epithelial ovarian cancers [10]. *F3* is one such gene [32], and is a major coagulation initiator. *F3* is up-regulated by both FLT1 and CP [32], [33]. Furthermore, F3 is inhibited by TFPI2 [39], which is down-regulated in our microarray. Other down-regulated genes that are inhibitors of coagulation include *THBD*, *PROCR*, *CD59*, and *PROS1* [40], [41].

More importantly, analysis of the CCOC data set revealed major activated pathways involved in angiogenesis and gycolysis. Both VEGF and its receptor, FLT1, were found to be up-regulated, as well as INHBA. INHBA is a member of the transforming growth factor-β superfamily that has been found to be up-regulated by VEGF [42]. Furthermore, it has been found to be an autocrine factor involved in tubulogenic morphogenesis by up-regulating VEGF and FLT1 [42], [43]. ADM is involved in angiogenesis and is transcriptionally up-regulated by HIF1α [44]. The dysregulation of genes involved in angiogenesis is likely multi-factorial, and our pathway analysis reveals cross-over with other pathways. NOTCH1 has been shown to up-regulate VEGF and SLC2A1 [34], [35]. The VEGF receptor, FLT1, has been shown to phosphorylate CRK and PLCG1, both of which are involved in cell motility[45]. Our results suggest one important protein in this pathway is HIF1α. The microarray suggests several mechanisms for activation of the HIF1α pathway in CCOC. MDM2 was found to be over-expressed, and it has been found to up-regulate HIF1α expression [46]. HSPCA stabilizes HIF1α protein, avoiding degradation via a VHL-independent pathway [47], and this gene was found to be over-expressed as well. SDHD is involved in HIF1α degradation [46], and was found to be under-expressed.

Multiple genes within glucose metabolism are up-regulated in CCOC. SLC2A1 is involved in glucose transport, and HK1/HK2 and ENO1/ENO2 are involved in glycolysis. All are known to be transcriptionally up-regulated by HIF1α [27], [28], [48]. HIF1α has been found to help protect cells against apoptosis, and this effect is mediated through the regulation of glucose transporters and glycolysis enzymes [28]. *In vivo* studies evaluating the tumoricidal effects of HIF1α disruption have demonstrated a greater correlation with glucose metabolism disruption [49], [50]. Over-expression of ENO1 and HIF1α in CCOC also demonstrates a clinical link to CCOC's association with endometriosis. Up to 50% of patients with endometriosis have been found to have autoantibodies to enolase [51], [52], possibly implying an immune response to overproduction of enolase. We demonstrated over-expression of ENO1 in both CCOC tumors and associated endometriotic lesions (Figure S3). Furthermore, recent literature has reported elevated HIF1α mRNA and protein levels in ectopic endometrial implants [53].

The relevant importance of these activated pathways are demonstrated in our *in vitro* and *in vivo* model systems. There is a major phenotypic difference between clear cell and serous cell lines in their rate of proliferation under *in vitro* conditions of HG. Knockdown of genes in either pathway, sensitizes the clear cell lines to HG conditions. This experiment suggests three major points. First, this suggests that a mechanism for the poorer prognosis for clear cell cancers when compared to the serous histotype, may be the ability of the cells to survive in the environment with limited oxygen and nutrients. Clear cell cancer cells were less affected by these stressful conditions when compared to serous cell lines. Second, disruption of the either angiogenesis or gycolytic pathways sensitizes these cells to these conditions, implying that they play a role in providing these cells with a survival advantage. Third, these pathways may serve as therapeutic targets.

The *in vivo* importance of the angiogenesis and glycolytic pathway for tumor growth was seen by systemic usage of small molecule inhibitors or small interfering RNA (siRNA) in nude mice. Since the microarray revealed mechanisms for activation of pathways of angiogenesis, we evaluated the antitumor activity of sunitinib. Sunitinib (SU11248; sutent), an antiangiogenic drug, was effective at inhibiting cellular proliferation and in combination with HIF1 α and enolase siRNA's indicated a synergistic antitumor activity, with a reduction in tumor nodule and tumor microvessel density.

To provide an additional level of validation, patient derived clear cell and serous ovarian cancer tumors were grafted under the renal capsule of NOD-SCID mice to mimic the clinically relevant cancers. This method allows high tissue perfusion and potentially rapid development of graft microvasculature. More importantly, the ovarian cancer tissues can be grown and serially transplanted under renal capsules of NOD-SCID mice, with minimal histological and genetic changes, and with retention of sensitivity to cytotoxic chemotherapy. These patient-derived cancer tissue lines are therefore very similar to the original cancer specimens and, as such, their xenografts in NOD-SCID mice provide ovarian cancer models that closely resemble the patients' malignancies [24], [25]. Sunitinib markedly reduced the growth and the microvessel density of the clear cell carcinoma xenografts, it did not significantly affect the tumor volume nor the microvessel density of any of the three serous carcinomas.

Based on our microarray and results of knockdown and patient derived tumor xenografts experiments, angiogenesis and glycolysis pathways appear to be important in the survival and progression of CCOC. The marked effect of sunitinib on the growth and viability of the clear cell xenografts as distinct from the serous carcinoma xenografts indicates that sunitinib is potentially useful for targeted therapy of CCOC. Our studies with combination therapy of sunitinib and RNAi demonstrate synergistic anti tumor activity in CCOC and these results provide a rational basis for specific therapy in these patients. Drugs targeting angiogenesis (antibodies or TKIs) along with ones which inhibit tumor metabolism (mTor inhibitors) would be one possible choice.

## Supporting Information

Figure S1 **Cell Cycling analysis of OVCA420 (serous) and ES-2 (clear cell).** After 48 hours of normal oxygen/normal glucose (NN) and hypoxia/glucose deprivation (HG), a cell cycling analysis was performed, demonstrating a significant increase in the both G2/M phase in only the OVCA420 cell line.
(DOC)

Figure S2 **Caspase assay for OVCA420. Caspase-3 activity was determined by relative fluorescence per µg protein (see methods section for details).**
(DOC)

Figure S3 **Immunohistochemical staining for ENO1.** Both endometriotic lesions (left) and associated clear cell ovarian tumors (right) strongly stain for enolase 1 (ENO1).
(DOC)

Table S1 **qRT-PCR analysis of randomly selected 12 genes and corresponding primer sequences.** * Student T-test P-value.
(DOC)

## References

[1] Schiller W (1939). Mesonephroma ovarii.. Am J Cancer.

[2] Genton CY (1979). Ultrastructure of clear cell carcinoma of the ovary. Case report and review of the literature.. Virchows Arch A Pathol Anat Histol.

[3] Silverberg SG (1973). Ultrastructure and histogenesis of clear cell carcinoma of the ovary.. Am J Obstet Gynecol.

[4] Jenison EL, Montag AG, Griffiths CT, Welch WR, Lavin PT (1989). Clear cell adenocarcinoma of the ovary: a clinical analysis and comparison with serous carcinoma.. Gynecol Oncol.

[5] Kennedy AW, Biscotti CV, Hart WR, Webster KD (1989). Ovarian clear cell adenocarcinoma.. Gynecol Oncol.

[6] Crozier MA, Copeland LJ, Silva EG, Gershenson DM, Stringer CA (1989). Clear cell carcinoma of the ovary: a study of 59 cases.. Gynecol Oncol.

[7] Kobel M, Kalloger SE, Huntsman DG, Santos JL, Swenerton KD (2010). Differences in tumor type in low-stage versus high-stage ovarian carcinomas.. Int J Gynecol Pathol.

[8] Sugiyama T, Kamura T, Kigawa J, Terakawa N, Kikuchi Y (2000). Clinical characteristics of clear cell carcinoma of the ovary: a distinct histologic type with poor prognosis and resistance to platinum-based chemotherapy.. Cancer.

[9] Pectasides D, Pectasides E, Psyrri A, Economopoulos T (2006). Treatment issues in clear cell carcinoma of the ovary: a different entity?. Oncologist.

[10] Matsuura Y, Robertson G, Marsden DE, Kim SN, Gebski V (2007). Thromboembolic complications in patients with clear cell carcinoma of the ovary.. Gynecol Oncol.

[11] Nordback I, Lauslahti K (1980). Clinicopathologic and histochemical study of ovarian clear cell carcinoma.. Int J Gynaecol Obstet.

[12] Dickersin GR, Welch WR, Erlandson R, Robboy SJ (1980). Ultrastructure of 16 cases of clear cell adenocarcinoma of the vagina and cervix in young women.. Cancer.

[13] Zorn KK, Bonome T, Gangi L, Chandramouli GV, Awtrey CS (2005). Gene expression profiles of serous, endometrioid, and clear cell subtypes of ovarian and endometrial cancer.. Clin Cancer Res.

[14] Schwartz DR, Kardia SL, Shedden KA, Kuick R, Michailidis G (2002). Gene expression in ovarian cancer reflects both morphology and biological behavior, distinguishing clear cell from other poor-prognosis ovarian carcinomas.. Cancer Res.

[15] Zorn KK, Jazaeri AA, Awtrey CS, Gardner GJ, Mok SC (2003). Choice of normal ovarian control influences determination of differentially expressed genes in ovarian cancer expression profiling studies.. Clin Cancer Res.

[16] Bonome T, Lee JY, Park DC, Radonovich M, Pise-Masison C (2005). Expression profiling of serous low malignant potential, low-grade, and high-grade tumors of the ovary.. Cancer Res.

[17] Donninger H, Bonome T, Radonovich M, Pise-Masison CA, Brady J (2004). Whole genome expression profiling of advance stage papillary serous ovarian cancer reveals activated pathways.. Oncogene.

[18] Aponte M, Jiang W, Lakkis M, Li MJ, Edwards D (2008). Activation of platelet-activating factor receptor and pleiotropic effects on tyrosine phospho-EGFR/Src/FAK/paxillin in ovarian cancer.. Cancer Res.

[19] Landen CN, Chavez-Reyes A, Bucana C, Schmandt R, Deavers MT (2005). Therapeutic EphA2 gene targeting in vivo using neutral liposomal small interfering RNA delivery.. Cancer Res.

[20] Halder J, Kamat AA, Landen CN, Han LY, Lutgendorf SK (2006). Focal adhesion kinase targeting using in vivo short interfering RNA delivery in neutral liposomes for ovarian carcinoma therapy.. Clin Cancer Res.

[21] Landen CN, Lin YG, Armaiz Pena GN, Das PD, Arevalo JM (2007). Neuroendocrine modulation of signal transducer and activator of transcription-3 in ovarian cancer.. Cancer Res.

[22] Ebos JM, Lee CR, Christensen JG, Mutsaers AJ, Kerbel RS (2007). Multiple circulating proangiogenic factors induced by sunitinib malate are tumor-independent and correlate with antitumor efficacy.. Proc Natl Acad Sci U S A.

[23] Sun L, Liang C, Shirazian S, Zhou Y, Miller T (2003). Discovery of 5-[5-fluoro-2-oxo-1,2- dihydroindol-(3Z)-ylidenemethyl]-2,4- dimethyl-1H-pyrrole-3-carboxylic acid (2-diethylaminoethyl)amide, a novel tyrosine kinase inhibitor targeting vascular endothelial and platelet-derived growth factor receptor tyrosine kinase.. J Med Chem.

[24] Lee CH, Xue H, Sutcliffe M, Gout PW, Huntsman DG (2005). Establishment of subrenal capsule xenografts of primary human ovarian tumors in SCID mice: potential models.. Gynecol Oncol.

[25] Press JZ, Kenyon JA, Xue H, Miller MA, De Luca A (2008). Xenografts of primary human gynecological tumors grown under the renal capsule of NOD/SCID mice show genetic stability during serial transplantation and respond to cytotoxic chemotherapy.. Gynecol Oncol.

[26] Mendel DB, Laird AD, Xin X, Louie SG, Christensen JG (2003). In vivo antitumor activity of SU11248, a novel tyrosine kinase inhibitor targeting vascular endothelial growth factor and platelet-derived growth factor receptors: determination of a pharmacokinetic/pharmacodynamic relationship.. Clin Cancer Res.

[27] Semenza GL, Jiang BH, Leung SW, Passantino R, Concordet JP (1996). Hypoxia response elements in the aldolase A, enolase 1, and lactate dehydrogenase A gene promoters contain essential binding sites for hypoxia-inducible factor 1.. J Biol Chem.

[28] Kilic M, Kasperczyk H, Fulda S, Debatin KM (2007). Role of hypoxia inducible factor-1 alpha in modulation of apoptosis resistance.. Oncogene.

[29] Kelly BD, Hackett SF, Hirota K, Oshima Y, Cai Z (2003). Cell type-specific regulation of angiogenic growth factor gene expression and induction of angiogenesis in nonischemic tissue by a constitutively active form of hypoxia-inducible factor 1.. Circ Res.

[30] Tsao MS, Liu N, Nicklee T, Shepherd F, Viallet J (1997). Angiogenesis correlates with vascular endothelial growth factor expression but not with Ki-ras oncogene activation in non-small cell lung carcinoma.. Clin Cancer Res.

[31] Nevo O, Soleymanlou N, Wu Y, Xu J, Kingdom J (2006). Increased expression of sFlt-1 in in vivo and in vitro models of human placental hypoxia is mediated by HIF-1.. Am J Physiol Regul Integr Comp Physiol.

[32] Cuadrado MJ, Buendia P, Velasco F, Aguirre MA, Barbarroja N (2006). Vascular endothelial growth factor expression in monocytes from patients with primary antiphospholipid syndrome.. J Thromb Haemost.

[33] Crutchley DJ, Que BG (1995). Copper-induced tissue factor expression in human monocytic THP-1 cells and its inhibition by antioxidants.. Circulation.

[34] Wang Z, Banerjee S, Li Y, Rahman KM, Zhang Y (2006). Down-regulation of notch-1 inhibits invasion by inactivation of nuclear factor-kappaB, vascular endothelial growth factor, and matrix metalloproteinase-9 in pancreatic cancer cells.. Cancer Res.

[35] Ciofani M, Zuniga-Pflucker JC (2005). Notch promotes survival of pre-T cells at the beta-selection checkpoint by regulating cellular metabolism.. Nat Immunol.

[36] Wu R, Hendrix-Lucas N, Kuick R, Zhai Y, Schwartz DR (2007). Mouse model of human ovarian endometrioid adenocarcinoma based on somatic defects in the Wnt/beta-catenin and PI3K/Pten signaling pathways.. Cancer Cell.

[37] Yamaguchi K, Mandai M, Oura T, Matsumura N, Hamanishi J (2010). Identification of an ovarian clear cell carcinoma gene signature that reflects inherent disease biology and the carcinogenic processes.. Oncogene.

[38] Mok SC, Bonome T, Vathipadiekal V, Bell A, Johnson ME (2009). A gene signature predictive for outcome in advanced ovarian cancer identifies a survival factor: microfibril-associated glycoprotein 2.. Cancer Cell.

[39] Sprecher CA, Kisiel W, Mathewes S, Foster DC (1994). Molecular cloning, expression, and partial characterization of a second human tissue-factor-pathway inhibitor.. Proc Natl Acad Sci U S A.

[40] Cappellini MD (2007). Coagulation in the pathophysiology of hemolytic anemias.. Hematology Am Soc Hematol Educ Program.

[41] Medina P, Navarro S, Estelles A, Espana F (2007). Polymorphisms in the endothelial protein C receptor gene and thrombophilia.. Thromb Haemost.

[42] Maeshima K, Maeshima A, Hayashi Y, Kishi S, Kojima I (2004). Crucial role of activin a in tubulogenesis of endothelial cells induced by vascular endothelial growth factor.. Endocrinology.

[43] Poulaki V, Joussen AM, Mitsiades N, Mitsiades CS, Iliaki EF (2004). Insulin-like growth factor-I plays a pathogenetic role in diabetic retinopathy.. Am J Pathol.

[44] Frede S, Freitag P, Otto T, Heilmaier C, Fandrey J (2005). The proinflammatory cytokine interleukin 1beta and hypoxia cooperatively induce the expression of adrenomedullin in ovarian carcinoma cells through hypoxia inducible factor 1 activation.. Cancer Res.

[45] Ito N, Huang K, Claesson-Welsh L (2001). Signal transduction by VEGF receptor-1 wild type and mutant proteins.. Cell Signal.

[46] Pollard PJ, El-Bahrawy M, Poulsom R, Elia G, Killick P (2006). Expression of HIF-1alpha, HIF-2alpha (EPAS1), and their target genes in paraganglioma and pheochromocytoma with VHL and SDH mutations.. J Clin Endocrinol Metab.

[47] Isaacs JS, Jung YJ, Mimnaugh EG, Martinez A, Cuttitta F (2002). Hsp90 regulates a von Hippel Lindau-independent hypoxia-inducible factor-1 alpha-degradative pathway.. J Biol Chem.

[48] Gwak GY, Yoon JH, Kim KM, Lee HS, Chung JW (2005). Hypoxia stimulates proliferation of human hepatoma cells through the induction of hexokinase II expression.. J Hepatol.

[49] Welsh S, Williams R, Kirkpatrick L, Paine-Murrieta G, Powis G (2004). Antitumor activity and pharmacodynamic properties of PX-478, an inhibitor of hypoxia-inducible factor-1alpha.. Mol Cancer Ther.

[50] Chen J, Zhao S, Nakada K, Kuge Y, Tamaki N (2003). Dominant-negative hypoxia-inducible factor-1 alpha reduces tumorigenicity of pancreatic cancer cells through the suppression of glucose metabolism.. Am J Pathol.

[51] Gitlits VM, Toh BH, Sentry JW (2001). Disease association, origin, and clinical relevance of autoantibodies to the glycolytic enzyme enolase.. J Investig Med.

[52] Walter M, Berg H, Leidenberger FA, Schweppe KW, Northemann W (1995). Autoreactive epitopes within the human alpha-enolase and their recognition by sera from patients with endometriosis.. J Autoimmun.

[53] Wu MH, Chen KF, Lin SC, Lgu CW, Tsai SJ (2007). Aberrant expression of leptin in human endometriotic stromal cells is induced by elevated levels of hypoxia inducible factor-1alpha.. Am J Pathol.

