# Supplementary material for: Identification of Novel Therapeutic Targets in Microdissected Clear Cell Ovarian Cancers
Source: PLoS One. 2011 Jul 6;6(7):e21121. doi: 10.1371/journal.pone.0021121 (PMC3130734; doi:10.1371/journal.pone.0021121)
Supplement: Figure S1 — Cell Cycling analysis of OVCA420 (serous) and ES-2 (clear cell). After 48 hours of normal oxygen/normal glucose (NN) and hypoxia/glucose deprivation (HG), a cell cycling analysis was performed, demonstrating a significant increase in the both G2/M phase in only the OVCA420 cell line. (DOC) [file pone.0021121.s001.doc]

**Supplementary Figure S1**.
