# Supplementary figures and images for: Identification of Novel Therapeutic Targets in Microdissected Clear Cell Ovarian Cancers
Source: PLoS One. 2011 Jul 6;6(7):e21121. doi: 10.1371/journal.pone.0021121 (PMC3130734; doi:10.1371/journal.pone.0021121)

**Supplementary Figure S2**


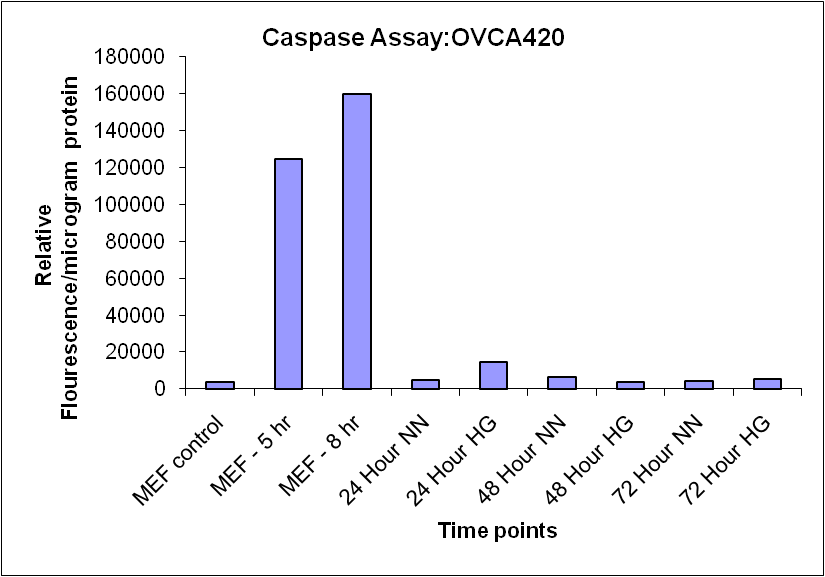

Supplement: Figure S2 — Caspase assay for OVCA420. Caspase-3 activity was determined by relative fluorescence per µg protein (see methods section for details). (DOC) [file pone.0021121.s002.doc]
