# Supplementary material for: Identification of Novel Therapeutic Targets in Microdissected Clear Cell Ovarian Cancers
Source: PLoS One. 2011 Jul 6;6(7):e21121. doi: 10.1371/journal.pone.0021121 (PMC3130734; doi:10.1371/journal.pone.0021121)
Supplement: Figure S3 — Immunohistochemical staining for ENO1. Both endometriotic lesions (left) and associated clear cell ovarian tumors (right) strongly stain for enolase 1 (ENO1). (DOC) [file pone.0021121.s003.doc]

**Supplementary Figure S3**
