# Supplementary material for: Identification of Novel Therapeutic Targets in Microdissected Clear Cell Ovarian Cancers
Source: PLoS One. 2011 Jul 6;6(7):e21121. doi: 10.1371/journal.pone.0021121 (PMC3130734; doi:10.1371/journal.pone.0021121)
Supplement: Table S1 — qRT-PCR analysis of randomly selected 12 genes and corresponding primer sequences. * Student T-test P-value. (DOC) [file pone.0021121.s004.doc]

**Supplementary Table S1.**

| **Gene** | **Fold Change** | **P-Value*** | **Forward** | **Reverse** |
| --- | --- | --- | --- | --- |
| *HBB* | -360.2 | 0.041 | TTGAGTCCTTTGGGGATCTG | CTTTCTTGCCATGAGCCTTC |
| *GAS1* | -42.6 | 0.005 | TCTGCTCCTATCAGGGTTGC | CAATGGACTGTGGGTTTTGA |
| *MNDA* | -323.1 | 0.035 | CAAGCAAGCATCTGGAACAA | AGCTTGCGGTCAACTGTTCT |
| *EFEMP1* | -202.20 | 0.034 | CCATGGTATAAAGTGGGCATT | ATGATGGCTGCCTCCTTATG |
| *MUC1* | 10.5 | 0.043 | GCTCACAGCCTCCTTCAGAG | GTTCAGGATCCCCGCTATCT |
| *PRC1* | 4.1 | 0.040 | GTTCCAATGGGTTGAGCTGT | AGGCAGACAGCGGAAGAATA |
| *TACSTD1* | 40.3 | 0.004 | TGCAGGGTCTAAAAGCTGGT | CCCTATGCATCTCACCCATC |
| *TOP2A* | 16.4 | 0.001 | TTCTTGATATGCCCCTTTGG | GCTTCAACAGCCTCCAATTC |
| *FLT1* | 3593.0 | 0.003 | GCAGGGACATGGAATTAAGGC | TGAGCTTTCTCTGCCCATTTTC |
| *TOP1* | 153.1 | 0.038 | CCACCTCCACAACGATTCC | GTTCACTGTTGCTATGCTTGG |
| *COL4A3* | 6.6 | 0.748 | TTACCAGCATACCCACACAAA | TCCGAGTCTGATAAACCAACAA |
| *ARPC5L* | 4 | 0.068 | TGCGGTGTGTTTCGTGTAT | CAGAGGGCCTGTGGATTG |
| Validation |  | 83.30% |  |  |
